# Supplementary figures and images for: High numbers of differentiated CD28null CD8+ T cells are associated with a lowered risk for late rejection and graft loss after kidney transplantation
Source: PLoS One. 2020 Feb 5;15(2):e0228096. doi: 10.1371/journal.pone.0228096 (PMC7001918; doi:10.1371/journal.pone.0228096)

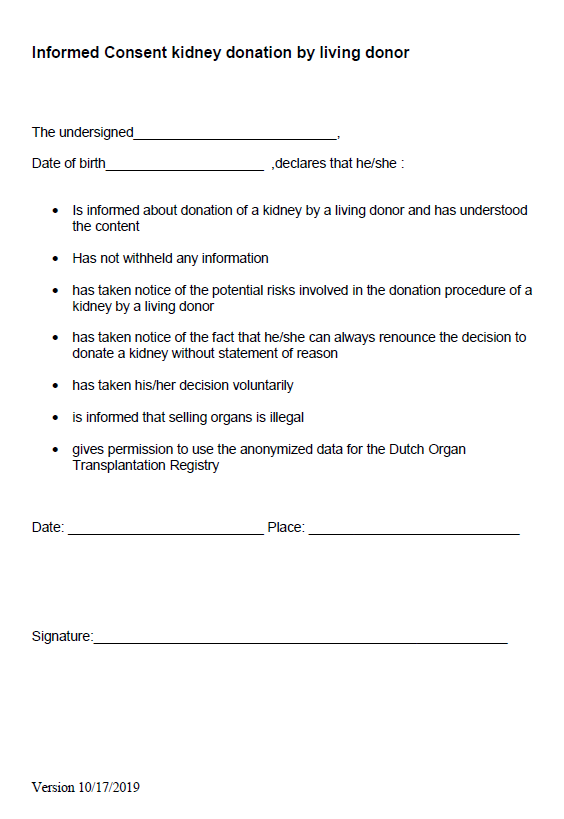

Supplement: S1 Fig — (TIF) [file pone.0228096.s002.tif]
