# Supplementary material for: High numbers of differentiated CD28null CD8+ T cells are associated with a lowered risk for late rejection and graft loss after kidney transplantation
Source: PLoS One. 2020 Feb 5;15(2):e0228096. doi: 10.1371/journal.pone.0228096 (PMC7001918; doi:10.1371/journal.pone.0228096)
Supplement: Attachment — Submitted filename: response to the reviewers.docx [file pone.0228096.s003.docx]

Dear Senthilnathan Palaniyandi,

Hereby I resubmit on behalf of all authors the manuscript entitled “High numbers of differentiated CD28null CD8^+^ T cells are associated with a lowered risk for late rejection and graft loss after kidney transplantation” for publication in PLOSone.

The issues raised by the editor and the reviewers are addressed in a point-by-point fashion as shown below.

We hope that the manuscript in its current form is acceptable for publication in PLOSone.

Sincerely,

Dr Michiel Betjes

PLOS ONE

Journal Requirements:

When submitting your revision, we need you to address these additional requirements.

1. Please ensure that your manuscript meets PLOS ONE's style requirements, including those for file naming. The PLOS ONE style templates can be found at <http://www.journals.plos.org/plosone/s/file?id=wjVg/PLOSOne_formatting_sample_main_body.pdf> and <http://www.journals.plos.org/plosone/s/file?id=ba62/PLOSOne_formatting_sample_title_authors_affiliations.pdf>

*We have changed the manuscript accordingly.*

2.  We note that your study involved tissue/organ transplantation. Please provide the following information regarding tissue/organ donors for transplantation cases analyzed in your study.

1. Please provide the source(s) of the transplanted tissue/organs used in the study, including the institution name and a non-identifying description of the donor(s).

*Eurotransplant in case of deceased donors kidneys and Erasmus MC for living donor kidney transplantations. This information is now provided in the revised manuscript.*

2. Please state in your response letter and ethics statement whether the transplant cases for this study involved any vulnerable populations; for example, tissue/organs from prisoners, subjects with reduced mental capacity due to illness or age, or minors.
- If a vulnerable population was used, please describe the population, justify the decision to use tissue/organ donations from this group, and clearly describe what measures were taken in the informed consent procedure to assure protection of the vulnerable group and avoid coercion.

- If a vulnerable population was not used, please state in your ethics statement, “None of the transplant donors was from a vulnerable population and all donors or next of kin provided written informed consent that was freely given.”

*A vulnerable population was not used and the required ethics statement is provided in the revised manuscript.*

3. In the Methods, please provide detailed information about the procedure by which informed consent was obtained from organ/tissue donors or their next of kin. In addition, please provide a blank example of the form used to obtain consent from donors, and an English translation if the original is in a different language.

*This procedure is now given in the Methods by referring to the legislation within the Eurotransplant region (Eurotransplant.org under legislation/eurotransplant) and the informed consent form for donors (translated in English) is included.*

4. Please indicate whether the donors were previously registered as organ donors. If tissues/organs were obtained from deceased donors or cadavers, please provide details as to the donors’ cause(s) of death.

*All deceased donor kidneys were allocated via Eurotransplant and donor registration was regulated per country according to national legislation. In our cohort, sixty percent of deceased donor kidneys were from deceased by cardiac death donors and 40% from deceased by brain death donors. This information is now given in the Methods section.*

5. Please provide the participant recruitment dates and the period during which transplant procedures were done (as month and year)

*The period during which the transplant procedures were performed is now provided in the revised manuscript.*

6. Please discuss whether medical costs were covered or other cash payments were provided to the family of the donor. If so, please specify the value of this support (in local currency and equivalent to U.S. dollars).

*All medical costs were covered by the medical insurance. This information is now provided in the revised manuscript.*

Additional Editor Comments (if provided):


[Note: HTML markup is below. Please do not edit.]

Reviewers' comments:

Reviewer's Responses to Questions

**Comments to the Author**

1. Is the manuscript technically sound, and do the data support the conclusions?

The manuscript must describe a technically sound piece of scientific research with data that supports the conclusions. Experiments must have been conducted rigorously, with appropriate controls, replication, and sample sizes. The conclusions must be drawn appropriately based on the data presented.

Reviewer #1: Yes

Reviewer #2: Yes

2. Has the statistical analysis been performed appropriately and rigorously?

Reviewer #1: No

Reviewer #2: Yes

3. Have the authors made all data underlying the findings in their manuscript fully available?

The [PLOS Data policy](https://eur01.safelinks.protection.outlook.com/?url=http%3A%2F%2Fwww.plosone.org%2Fstatic%2Fpolicies.action%23sharing&data=02%7C01%7Cm.g.h.betjes%40erasmusmc.nl%7Cf0f10716246e43b7088a08d750772ec5%7C526638ba6af34b0fa532a1a511f4ac80%7C0%7C1%7C637066345128719712&sdata=p44YvlIZNigpIu8hZqfES3zYKZbK4g2kvO%2FgRcUUdoY%3D&reserved=0) requires authors to make all data underlying the findings described in their manuscript fully available without restriction, with rare exception (please refer to the Data Availability Statement in the manuscript PDF file). The data should be provided as part of the manuscript or its supporting information, or deposited to a public repository. For example, in addition to summary statistics, the data points behind means, medians and variance measures should be available. If there are restrictions on publicly sharing data—e.g. participant privacy or use of data from a third party—those must be specified.

Reviewer #1: Yes

Reviewer #2: Yes

4. Is the manuscript presented in an intelligible fashion and written in standard English?

PLOS ONE does not copyedit accepted manuscripts, so the language in submitted articles must be clear, correct, and unambiguous. Any typographical or grammatical errors should be corrected at revision, so please note any specific errors here.

Reviewer #1: Yes

Reviewer #2: Yes

5. Review Comments to the Author

Please use the space provided to explain your answers to the questions above. You may also include additional comments for the author, including concerns about dual publication, research ethics, or publication ethics. (Please upload your review as an attachment if it exceeds 20,000 characters)

Reviewer #1: In this study the authors characterized the T cell population prior to kidney transplantation in a cohort of 365 patients who received a kidney transplant form a living donor in the period 2007-2013 with the hypothesis that parameters indicative of an aged T-cell population associate with a lower risk for late rejection.
They found that the number and percentage of differentiated T cells were lower in patients with biopsy-proven late rejection (n. 50, diagnosed at a median of 44 months) and were independently and inveresely associated with rejection occurrence.
The study is well-conducted and the main strength is that a relatively large and homogeneous cohort of patients was analysed, with a relatevely long follow-up (median 70-80 months).

In my opinion, there are some points to be elucidated:

- were all the biopsies performed on clinical indication? *Yes*

- was there a correlation between the T cell population before the transplantation and the type of rejection? *No*

- The data about DSA pre-transplantation is missing; this would l be an important information in the evaluation of the immunological status of the recipients. Most of the rejection (39/50) were categorized as ABMR: were DSA searched at the time of the kidney biopsy? If so, it would be appropriate to evaluate the correlation between DSA and T cell population before the transplantation, and the association of DSA with graft loss.

*We agree with this comment and data about DSA pre-transplantation and of most patients at time of biopsy would be of value. Unfortunately, this information is largely absent as DSA were not structurally assessed before and at time of rejection within the time period of this study. This is stated in the manuscript.*

- in the Kaplan-Meier curves (Fig 1A, 1B and 2) the number of patients at risk should be reported

*This is now provided in the KM curves in the revised manuscript.*

- In the Kaplan-Meier analysis for late rejection-related graft loss (Fig 2) the time considered should be the time after the rejection diagnosis, not the time after transplantation

*The research question is whether pre-transplant numbers of CD28null T cells are related to late rejection and rejection-related graft loss. Therefore, we considered time after transplantation to graft loss. If we would take time after rejection to graft loss then we would study the question whether the numbers of CD28null T cells are associated with progression to graft loss* ***after rejection is diagnosed.*** *This is obviously a different approach and would test the hypothesis that CD28null T cells can mitigate or worsen the clinical course of rejection. We did this analysis but could not find such an effect.*

- A multivariate analysis for graft loss would be needed

*We now have included the results of the multivariate analysis for rejection-related graft loss in the results section of the revised manuscript.*

- page 11, line 176 should be "....had a significantly HIGHER percentage s of naive CD4 and CD8 T cells and LOWER percentages of the CD8 effector..."
- page 11, line 178 should be: "...corresponded with a significant LOWER percentage of CD28null CD8 T cells.."

*These errors have now been corrected.*

Reviewer #2: This is a retrospective study in kidney transplant recipients to determine if there is an association between T cell telomere length and T cell subset analysis (CD28 and CD8) correlated with late (>6 months) acute rejection. Several questions arise:

1. The overall population is of relatively immunologic risk - unsensitized, living donors. Despite this, the "late rejection" group had a very high early acute rejection rate -33%. Additionally, nearly 20% of the entire population had a late acute rejection. Can you explain the very high rate of early and late acute rejection in this low risk group

*The group of recipients has a mixed profile for their immunological risk as panel reactive antibodies were present in some recipients (either at time of transplantation or historically) and re-transplantations were performed. The % of early rejection diagnosed within the whole population is not that different from data obtained from registries which is always higher than most clinical trials with study drugs including low-immunological risk patients only.*

*For instance, in a recent paper from the ANZDATA registry the % early AR was 21% in a large cohort of first kidney transplantations and a significant risk factor for late rejection (Clayton et al*, [J Am Soc Nephrol.](https://www.ncbi.nlm.nih.gov/pubmed/31308074) *2019 Sep;30(9):1697-1707).*

*The percentage of late rejections (which is not synonymous with acute rejection) is a cumulative percentage obtained at a rather long follow-up. Patients transplanted at our center have their regular visits at their referral medical center but are once-yearly seen by a transplantation-nephrologist in our center. When a steady decline in renal allograft function is observed we perform a diagnostic kidney biopsy to rule out late (chronic) rejection as a cause. In this way we try to diagnose all cases of late rejection which yields a much higher number of late rejections than most studies with a low % of diagnostic biopsies late after transplantation (e.g. Naesens et al, Transplantation. 2014 Aug 27;98(4):427-35)*

2. No data is supplied regarding immunosuppressive levels at any time point post-tx. Given the very high rejection rates, this is an important variable that must be accounted for in your multi-variant analysis.

*This is a very difficult question to address. The large majority of patients were given tacrolimus in combination with MMF and prednisone within the first 6 months after transplantation with through levels for tacrolimus by protocol. This protocol is now mentioned within the revised manuscript. Thereafter the data on through levels are incomplete as most patients were seen in their referral nephrology center and combinations of immune suppressive drugs could be changed. In addition, emerging data strongly suggest that the degree of intra-individual variability is a much stronger predictor of graft survival than through levels only. In a recent paper we did analyze the through levels and IPV in patients with late humoral rejection in great detail (Sablik et al, Transplant Int* *31: 900-908, 2018 ) and found that the average tacrolimus through level was 6 which is above the general recommended through level of 5 ug/ml.*

3. There is a very high rate of anti-body mediated rejection compared to cell mediated rejection. Please explain

*Late rejection is frequently caused by anti-body mediated rejection and in the years after transplantation the incidence of cell mediated rejection becomes (very) low.*

4. Do you have data on development of donor specific antibody formation.

*Unfortunately, we have only data on DSA in the minority of patients before and after kidney transplantation as DSA assessment was not routinely performed. This is stated in the manuscript.*

5. Nearly 40% did not receive any induction therapy. How was induction therapy chosen?

*Within the study period, the use of anti-CD25 antibody was not yet the standard induction therapy. Induction with anti-CD25 antibody or a T cell depleting agent was done when the risk for acute rejection was thought to be high (e.g. high %PRA, high number of MM, frequent rejections in the past or with a repeated mismatch with a previous transplant).*

6. Can you expound on why telomere length had no influence on long-term outcomes?

*Telomere length is assessed on the whole populations of CD4 and CD8 T cells. It has a significant but variable relation with the recipient age and therefore is a “bulk” marker of immunological ageing. The association of an expanded CD28null T cell population and less rejection seems to be more cell specific and, although speculative, may indicate a role of e.g. suppressor CD8 T cells. This has been stated in the discussion of the manuscript.*

While revising your submission, please upload your figure files to the Preflight Analysis and Conversion Engine (PACE) digital diagnostic tool, [https://pacev2.apexcovantage.com/](https://eur01.safelinks.protection.outlook.com/?url=https%3A%2F%2Fpacev2.apexcovantage.com%2F&data=02%7C01%7Cm.g.h.betjes%40erasmusmc.nl%7Cf0f10716246e43b7088a08d750772ec5%7C526638ba6af34b0fa532a1a511f4ac80%7C0%7C1%7C637066345128729704&sdata=gzXWl%2B%2FlJ53VxTiCVoE3XXawmNlzK749rA%2BOTNKmRWU%3D&reserved=0). PACE helps ensure that figures meet PLOS requirements. To use PACE, you must first register as a user. Registration is free. Then, login and navigate to the UPLOAD tab, where you will find

*This has been performed.*
