# Supplementary material for: High numbers of differentiated CD28null CD8+ T cells are associated with a lowered risk for late rejection and graft loss after kidney transplantation
Source: PLoS One. 2020 Feb 5;15(2):e0228096. doi: 10.1371/journal.pone.0228096 (PMC7001918; doi:10.1371/journal.pone.0228096)
Supplement: Attachment — Submitted filename: rebuttalPlOs.docx [file pone.0228096.s004.docx]

Dear editor,

The comments of reviewer #2 are adressed in a point-by-point fashion as shown below.

We hope the manuscript is now suitable for publication.

Sincerely,

Dr Michiel Betjes

I appreciate your response to both reviewers. Several questions still exist:

1. It is still not clear why there is a high rate of acute rejection is a very low immunological risk population. The overwhelming majority of recipients were unsensitized, living donor recipients. Acute rejection rates of 19.5% at 6 months in this group is still far higher than data from the Europenan EKiTE registry (Lorent, M., Foucher, Y., Kerleau, K. et al. The EKiTE network (epidemiology in kidney transplantation - a European validated database): an initiative epidemiological and translational European collaborative research. BMC Nephrol 20, 365 (2019) doi:10.1186/s12882-019-1522-8) showing a 79% 2 year rejection free in a population consisting of 81% deceased donors with a large portion that were sensitized or repeat transplants.

*We agree with the reviewer that in our patient population a relatively high rate of acute rejection is observed. The reasons are difficult to identify as we use a triple immune suppressive regimen including prednison/MMF/tacrolimus with adequate through levels defined by protocol (see M&M). Comparing patient populations is always difficult with potential unrecognized bias. For instance, the EKiTE data show T cell depletion as induction therapy in 37% of recipients while we rarely give this kind of induction therapy in the Netherlands. In other countries like the USA this percentage can be as high as 70% in some centres. On the other hand, publications from Japan and Korea show a very low AR percentage without T cell depletion and a standard IS regimen.*

*Virtually all our episodes of rejection are biopsy proven. In other studies, underreporting of rejection may occur as no biopsy was done before anti-rejection therapy. Our kidney biopsies are scored by a board-certified renal pathologist but we cannot rule out the possibility that our pathologists are leaning more to a diagnosis of rejection (for instance in cases of borderline acute rejection) than their colleagues in other centres.*

*In summary, we cannot satisfactorily address this comment but can only speculate on possible explanations. Of note, despite a relatively high AR rate the one-year graft and patient survival of our living donor kidney transplant program is excellent (>98%).*

2. How was the diagnosis of "late" acute rejection made? You mention that late rejections are not synonymous with acute rejection

*Late rejections are by definition occurring 6 months (admittedly our definition but a commonly used definition) after kidney transplantation. A rapid decline in renal function with a biopsy showing little chronicity but predominantly an influx of immune cells occurring many months after transplantation is compatible with an acute rejection and can be observed when e.g. patients are significantly under immune supressed. However, in most cases there is a progressive decrease in eGFR loss over a period of weeks to months with a biopsy showing rejection with signs of chronicity.*

3. You note that late rejections are most often antibody mediated but you have limited DSA data. Was the diagnosis of AMR made exclusively on the basis of C4D?

*All our renal biopsies were evaluated by an experienced renal pathologist who scores >150 renal transplant biopsies each year in our centre. The diagnosis of AMR was based on using the Banff criteria but C4D staining can be absent. However, a renal biopsy with all histological hallmarks of AMR was diagnosed as AMR after discussing the case with the nephrologists at our weekly renal pathology meeting. The Banff 2015 allows for this by having a category of suspicious ABMR when DSA and/or C4d are negative while all other criteria are met.*

4. The lack of immunosuppressive drug levels is a significant shortcoming

*We have a protocol in place for through levels of MMF and tacrolimus and at our centre we adhere quite strictly to this protocol as outlined in the M&M section. However, patients are eventually referred to their own nephrologist for follow-up and we cannot account for this period. Single through levels are not that reliable and there is a growing body of evidence that high intrapatient variability of tacrolimus through levels is actually more predictive of future rejection and graft loss. Unfortunately, we do not have these data but also cannot think of a reason why IPV could give a structural bias in our data (that is consistently more IPV in a subgroup of patients with high or low numbers of CD28null T cells).*

5. The selection of induction agents remains unanswered. The choice of anti-DC25 or ATG is a very different class of agents vs IL2-RA. The immunologic effect can be very different on your T cell subsets. Have you analyzed these groups separately to determine if there is any difference in outcomes.

*We are not sure what to answer. A very low number of patients received ATG induction and this is to small a group for meaningful statistical analysis. Anti-CD25 is synonymous with IL-2-receptor antagonist.*

*Within a multivariate analysis, the use of basiliximab (anti-CD25) was not a significant variable associated with late rejection. All these date are shown in table 1.*

*Within the discussion we recognize that the results obtained are possibly not generalizable when other IS drug regimes are used, notably induction with T cell depleting agents.*
